# Development and Initial Validation of a Scale to Measure Cognitive Demands of Flexible Work

**DOI:** 10.3389/fpsyg.2021.679471

**Published:** 2021-09-10

**Authors:** Roman Prem, Bettina Kubicek, Lars Uhlig, Vera Baumgartner, Christian Korunka

**Affiliations:** ^1^Institute of Psychology, Faculty of Natural Sciences, University of Graz, Graz, Austria; ^2^Department of Occupational, Economic and Social Psychology, Faculty of Psychology, University of Vienna, Vienna, Austria

**Keywords:** cognitive demands, flexible work, scale development, validation, structuring of work tasks, planning of working times, planning of working places, coordinating with others

## Abstract

With globalization, digitalization, and the spread of information and communication technologies, rules regulating work have been softened or completely abolished. Consequently, employees face additional cognitive demands to plan, structure, and coordinate their work. To capture these demands of contemporary work, we constructed and initially validated the Cognitive Demands of Flexible Work (CODE) scale. The scale comprises four subscales (i.e., structuring of work tasks, planning of working times, planning of working places, and coordinating with others). We initially validated the scale in three independent studies (overall *N* = 1,129) in German and English. Confirmatory factor analyses supported the four-factor structure, as well as scalar invariance, of the different language versions. Moreover, the subscales showed convergent and divergent validity with related constructs such as requirements for problem solving or autonomy. The criterion validity for emotional exhaustion, engagement, positive work rumination, negative work rumination, and problem-solving pondering suggested that cognitive demands of flexible work can be construed as challenge demands. However, relationships with emotional exhaustion were not significant. Overall, the CODE scale was shown to be a reliable and valid instrument to measure cognitive demands of flexible work.

## Introduction

The organization of work and the associated working conditions change according to societal and technological developments (Allvin and Movitz, 2017; Flecker et al., 2017). Over the last decades, globalization, individualization, and digitalization have changed working life and resulted in increasingly flexible work environments in terms of when, where, and how work is conducted (Putnam et al., 2014). Moreover, managerial strategies have shifted the responsibility for regulating work from management to employees (Allvin et al., 2011). These developments have led to additional cognitive demands for employees: With more flexibility and responsibility, cognitive demands for structuring tasks arise. In addition, the opportunity to work flexibly in terms of time and place (e.g., Wessels et al., 2019) leads to cognitive demands for planning when and where work is conducted. Furthermore, the need to arrange work with coworkers and clients who may not be present at the same time and place entails cognitive demands for coordinating with others.

Despite the increasing prevalence of cognitive demands of flexible work, there is still no measure that captures the extent of these specific demands to which employees are exposed. Established scales are either too unspecific or have a different focus. For example, the Work Design Questionnaire (WDQ; Morgeson and Humphrey, 2006), one of the most comprehensive measures of work characteristics, covers three facets of autonomy, namely freedom in (1) work scheduling, (2) decision-making, and (3) work methods. However, these refer to *decision possibilities* and do not measure *decision requirements* and the specific cognitive demands that stem from such requirements (Frese and Zapf, 1994; Allvin et al., 2011). The WDQ (Morgeson and Humphrey, 2006) also measures initiated and received interdependence but not the demands for coordination with others. Similarly, established measures of flexible work (e.g., Shockley and Allen, 2007) focus either on the availability or use of temporal and spatial flexibility. These measures do not capture the demands for work-time and workplace planning or coordinating work with others that may result from workplace flexibility (Allvin et al., 2011).

Some existing scales deal more specifically with flexible or deregulated work, but these scales have different foci and usually cover other aspects of changing working conditions, such as demands for self-directed career development, learning, and effort regulation. The Intensification of Job Demands Scale (Kubicek et al., 2015), for example, captures intensified job-related planning and decision-making demands. The focus, however, is on the intensification of these demands over a longer period as a result of social acceleration. The Flexibility Requirements Scales (Höge, 2011) focus on individually perceived expectations that an organization conveys to its employees regarding self-directed behavior related to flexibility but not on cognitive demands that could result from these expectations. For example, the requirements for temporal flexibility subscale includes items regarding the employer's expectation that employees work overtime or are flexible as far as their working hours are concerned rather than the cognitive demands of planning one's working times. Finally, the Job Design Demands Scale (Dettmers and Bredehöft, 2020) touches on the idea that more autonomous working conditions lead to additional demands for employees that include structuring and organizing one's work on one's own. This can be regarded as an important first step in providing measures that capture the cognitive demands related to modern working conditions. However, the Job Design Demands Scale does not include measures that focus on planning working times and working places or coordinating with others in flexibly organized work.

Given this lack of available instruments, the goal of the present study is to develop and initially validate a measure of cognitive demands of flexible work. Such a measure is needed because with the changing nature of work, different working conditions prevail, and cognitive demands have become an important feature of flexible work that—to varying degrees from job to job—is relevant to many modern workplaces. An instrument that captures the cognitively demanding aspects of flexible work can shed light on the processes that link flexible work with negative outcomes in employees and help to explain findings on negative or neutral effects of flexible work on employee well-being (Ter Hoeven and van Zoonen, 2015). Moreover, the instrument could help to advance scholarly knowledge about the extent of these demands in today's work settings and improve our understanding of the adverse and favorable effects of modern working conditions on employees' well-being, motivation, and learning (Grant et al., 2011). Without a reliable and valid measure, we are unable to provide a solid empirical basis to integrate these demands into established theoretical models such as the job demand—control model (Karasek and Theorell, 1990), the job demands—resources theory (Bakker and Demerouti, 2017), or the challenge—hindrance stressor framework (LePine et al., 2005).

To advance research and theory on contemporary working conditions, we sought to develop a measure that we call the Cognitive Demands of Flexible Work (CODE) scale. To validate the scale, we will assess construct validity and reliabilities and show that the factor structure of the CODE subscales is the same across German and English versions. We will further test convergent and divergent validity with other job-related factors, as well as criterion validity with work outcomes. Finally, we will investigate whether the means of the CODE subscales differ between groups of employees that should be exposed differently to flexible work environments.

## Cognitive Demands of Flexible Work

The last decades have brought significant growth in globalization and global operations (Castells, 2010), as well as enhanced use of information technology (Cascio and Montealegre, 2016). As a consequence of these transformations, work has become more flexible in various respects reflected in changes in the regulatory conditions of work. According to Allvin et al. (2011), “rules and norms that have traditionally defined and directed work, in various manners and to varying degrees, are now being softened up or simply abolished” (p. 218).

Although the term “flexible work” is sometimes used to refer to less-regulated ways of how labor is contractually organized (e.g., crowd work, gig work, and contingent work), we will focus in the following on the aspects of flexible work that reflect deregulated working conditions in terms of how work is conducted. Many employees in today's companies and organizations are exposed to different forms of flexible work, such as flexible working hours, telework (e.g., DiMartino and Wirth, 1990), and an increased projectification of organizations (e.g., Midler, 1995).

Overall, a growing number of jobs can be described as low-regulated or unregulated, in the sense that employees are expected to organize and plan their work more or less on their own. For example, in flextime and flexplace arrangements, the time and place of work are no longer solely determined by the organization. Further, with the increased use of projects and project-like work forms (e.g., agile forms of work organization), there has been a shift from standard operating procedures to goal-oriented performance, where work activities and coordination with others are also no longer determined only by the organization.

Such deregulation of work does not necessarily mean that control in organizations has become irrelevant. Rather, employees, instead of management, are required to take control. Thus, control has become increasingly indirect by being transferred to the employees (Flecker et al., 2017). This shift from direct control toward indirect control has created flexibility regarding how, when, where, and with whom people work. In addition, it has increased employees' responsibility for regulating their work (Allvin et al., 2011).

Although flexibility is associated with more discretion for employees (Gajendran and Harrison, 2007), it also puts additional cognitive demands on them because it increases the decisions to be made (Allvin et al., 2013; Höge and Hornung, 2015; Dettmers and Bredehöft, 2020). In flexibly organized work, employees not only receive more decision latitude but are oftentimes also required to use it efficiently, therefore facing additional job demands. In a way, flexible work entails aspects of job demands and job control (cf. Karasek and Theorell, 1990). Although choice is important for people and allows us to control our lives, having more choices is not always beneficial. As Schwartz (2016) points out in his seminal book *The Paradox of Choice*, a variety of options can overburden people and take up time and energy that should be devoted to other matters. In this sense, flexible work creates new demands for employees to self-organize and self-regulate their work activities (Pongratz and Voß, 2003; Allvin et al., 2013; Höge and Hornung, 2015). With fewer directives from management and higher individual responsibility to regulate work, employees must devote time and energy to structuring, planning, and coordinating their work. In line with this argument, chronic flextime use has been shown to require high levels of self-organization and coordination because employees cannot rely on an established daily temporal routine (Spieler et al., 2017). Moreover, initial evidence suggests that at least some of the demands that more flexible and autonomous work entails could also help to explain the adverse effects on burnout and job-related cognitions during leisure time (Dettmers and Bredehöft, 2020).

To conceptualize cognitive demands of flexible work, we rely on the areas of flexible or deregulated work that Allvin et al. (2011) put forward: performance, time, place, and social relations. Hence, we suggest that cognitive demands of flexible work entail structuring work tasks, planning (working times and working places), and coordinating work with others. Such cognitive demands of flexible work require mental effort to process information, solve problems, and make decisions regarding ordering work tasks and monitoring work progress, scheduling when and where work tasks should be dealt with, and coordinating with supervisors, coworkers, and clients. In the following we will describe each sub-dimension of cognitive demands of flexible work in more detail.

### Structuring of Work Tasks

With the deregulation of work, the logic of corporate labor control has changed. Direct control, characterized by work tasks and the working order that stipulate the process of work being defined and prescribed by management, is reduced, and requirements for autonomous planning and structuring work increase (Pongratz and Voß, 2003; Kubicek et al., 2015). Employees are increasingly expected to take a self-directed and outcome-oriented approach in fulfilling their tasks and achieving their work goals (Höge and Hornung, 2015). Consequently, employees must individually define, structure, and take responsibility for their performance and the completion of work tasks (Allvin et al., 2011; Dettmers and Bredehöft, 2020). Cognitive demands for structuring work refer to requirements on the part of the employee to not only define individual work steps but also determine the ordering of the work steps and monitor the progress of work.

### Planning of Working Times

The planning of working times concerns the temporal aspects of work arrangements. With the increase in temporal flexibility, fixed working hours are less common. This trend is evident, for example, in the decline of classic “nine-to-five jobs” in favor of flexible and trust-based working hours (Höge and Hornung, 2015). In such working arrangements, employees must individually decide and plan when, for how long, and how much they work (Hill et al., 2008). This not only includes deciding the start and end points of a working day but also includes being able to adjust the length of the working day. Often, employees have to keep track of their working hours and are expected to offset overtime and missing hours over longer periods while adjusting to workload demands. Thus, temporal flexibility increases the number of decisions that employees must make (Allen et al., 2013). Hence, cognitive demands for work time planning include having to decide on one's own when to start, pause, and end one's workday, how long one works on workdays, and when one plans to take breaks.

### Planning of Working Places

In analogy with work time planning, the planning of working places concerns the spatial aspect of work arrangements. With advances in information and communication technologies, organizations have moved from using traditional offices with permanent workplaces to more flexible forms of working that enable many employees to work from different working places inside (e.g., project rooms, open office space, or silent areas, e.g., Wohlers and Hertel, 2017) and outside the employers' premises (e.g., working from home, co-working spaces, or on the go). In the absence of a predefined workplace, employees have to decide individually where to work and which work venue best fits their tasks and needs (Wessels et al., 2019). Cognitive demands for workplace planning thus entail planning and deciding where to work on certain tasks, ensuring that all relevant work materials are available, and adjusting the workplace to the specific requirements of the task and individual needs.

### Coordinating With Others

In traditional and bureaucratically ruled organizations, cooperation between individuals is more or less specified by their organizational positions and facilitated through shared working hours and places. In flexible work, however, conditions for cooperation are to a large extent the responsibility of the individual (Allvin et al., 2011). Instead of relying on a given group of colleagues and a formal hierarchy, employees must establish and maintain the social contacts necessary for their work themselves and must coordinate their work with others. They must independently initiate and organize cooperation with the people needed to carry out the task at hand. This requirement may even become more demanding as colleagues and supervisors also work flexibly, which reduces shared working hours and places and makes spontaneous communication less likely (Hinds and Mortenson, 2005). Hence, cognitive demands for coordinating work with others entail having to agree upon work schedules, as well as the procedures and approaches needed to accomplish work tasks.

## Study 1: Scale Development, Reliabilities, and Construct Validity

The goal of the first study was to develop a scale to operationalize cognitive demands of flexible work that require employees to structure their work tasks, plan their working times and working places, and coordinate with others. Further, this study aimed to gather information on the construct validity of the newly developed scale.

To get a better understanding of how cognitive demands to structure, plan, and coordinate manifest in employees' working lives, we initially conducted four focus groups with employees working in flexible work environments. We asked participants to identify aspects of flexible work that they perceived as demanding. The focus group participants were working at different office locations of a large multi-national corporation that offered flexible working times and the option to work from home and in which employees were often involved in various projects with changing teams. The participants were recruited via a contact person from the human resource department. Participation was voluntary, and a small donation was made to a local charity project for each participant. Each group consisted of five to eleven employees with and without leadership responsibilities. At the end of each focus group, the results were summarized and clustered by a trained moderator and discussed again within the group.

Based on the information from the focus groups and the literature, we developed a pool of potential items. Because we intended to measure demands from the job rather than degrees of latitude or autonomy, we made sure that items were worded in terms of requirements (e.g., “My job requires me to …” or “At work, I have to …”). All items were positively worded to avoid artificial factor solutions, which could occur when including negatively worded items alongside positively worded items within the same scale (Dalal and Carter, 2015). Overall, we initially created an item pool of 24 items (i.e., 6 items for each of the 4 subscales) that was formulated in German.

We expected that all four subscales of the CODE scale would share positive relationships with other cognitive demands, such as information processing and problem solving (Hypothesis 1). Information processing at work refers to requirements to monitor and process data or other information in general, whereas problem solving is characterized by more specific requirements to actively process information, come up with ideas and solutions to solve non-routine problems, and deal with errors (Morgeson and Humphrey, 2006). Structuring, planning, and coordinating work will, in most cases, require action regulation at the intellectual level (cf. Zacher and Frese, 2018). Therefore, employees confronted with cognitive demands of flexible work should more often be required to monitor and process information about the progress of their work, how their schedule and work venue fit their work tasks, and how their work is aligned with that of their coworkers and other people at work. Moreover, when identifying discrepancies or inconsistencies, they will have to actively solve problems and come up with solutions.

Moreover, we expected that the subscale for the structuring of work tasks would be positively related to decision-making autonomy and work methods autonomy (Hypothesis 2). Both decision-making autonomy and work methods autonomy can be described as facets of general job autonomy that reflect how much freedom, independence, and discretion employees have at work (Morgeson and Humphrey, 2006). We assumed that possibilities for employees to make decisions as part of their work and/or in choosing their work methods would be necessary for cognitive demands related to the structuring of work to arise.

Further, we expected that the subscale for the planning of working times would be positively related to work scheduling autonomy and the availability of flextime (Hypothesis 3). Work scheduling autonomy is also considered an aspect of general job autonomy (Morgeson and Humphrey, 2006), whereas flextime refers to flexibility in the timing of work typically characterized by the possibilities of employees to vary the start and end times, as well as the length, of their workdays (Shockley and Allen, 2007; Kattenbach et al., 2010). We assumed that it would be necessary for employees to have autonomy regarding their work scheduling and/or to be provided with flextime possibilities for cognitive demands regarding the planning of working times to arise.

Similarly, we expected that the subscale for the planning of working places would be positively related to the availability of telework possibilities to work from home and the availability of telework possibilities to from other locations outside the employer's premises (Hypothesis 4). Both working from home and telework from other locations can be considered aspects of flexplace that describe alterations in the physical boundaries around work (Munsch et al., 2014; Thompson et al., 2015). We refer to working from home as the possibility to work from one's place of residence, whereas telework from other locations is considered working from locations other than one's place of residence that are outside the employers' premises (e.g., mobile telework). We assumed that for cognitive demands relating to the planning of workplaces to arise, it would be necessary for at least some spatial flexibility to be available to employees.

Finally, we expected that the subscale for coordinating with others would be positively related to the interdependence of tasks at work (Hypothesis 5). Interdependence can be described as the extent to which a job is connected with that of others. It is oftentimes differentiated into initiated interdependence, i.e., the extent to which others depend on one to finish one's work, and received interdependence, i.e., the extent to which one's work depends on others to finish their work (Morgeson and Humphrey, 2006). We assumed that interdependence would be necessary for cognitive demands with regard to coordinating with others to arise.

### Method

#### Procedure

Data on the German CODE scale were collected using an online questionnaire. The procedure and materials of this study did not undergo examination by an ethics committee, as the measures and procedures of our study followed standard procedures in applied psychological research, and we did not touch on sensitive topics (e.g., sexual orientation). Our protocol fully complied with the ethical principles of psychologists and the code of conduct of the American Psychological Association (2017). Participation was voluntary, and participants were free to withdraw whenever they wanted.

#### Sample

The sample consisted of employees from Germany working at least 30 contractual hours per week that were accessed via an ISO 26362-certified online panel (www.respondi.com). This panel recruits its participants via offline and online methods and ensures high quality through minimizing participation frequency and focusing on intrinsic motivation instead of financial dependency.

In total, 303 employees participated in study 1. About half (51.5%) of the sample was female. The average age of the participants was 43.13 years (*SD* = 11.11); they worked on average 41.70 h per week (*SD* = 6.09) and had worked for an average of 11.37 years (*SD* = 10.11) for their current company. About half (49.2%) of the participants held an academic degree.

#### Measures

Participants responded to the initial set of 24 items generated for the CODE scale. They were asked to indicate on a 5-point scale how the statements of the generated items applied to their work in general (1 = *not at all* to 5 = *fully*).

Most job-related factors for evaluating construct validity were measured using the German translation of the WDQ of Stegmann et al. (2010). *Information processing* and *problem solving* were each measured with 4 items, and *decision-making autonomy, work methods autonomy, work scheduling autonomy, initiated interdependence*, and *received interdependence* were each measured with 3 items. All WDQ items were measured on 5-point rating scales (1 = *strongly disagree* to 5 = *strongly agree*). The English version of the items can be found in the Appendix of Morgeson and Humphrey (2006).

*Availability of flextime* was measured with two self-created items (“I can decide on my own when to start and end a working day” and “I can decide on my own how many hours I work on a given day”) on a 7-point rating scale (1 = *does not apply at all* to 7 = *completely applies*). As both items were highly correlated (*r* = 0.85), we combined them to create a score for the availability of flextime.

*Availability of working from home* and *availability of telework from other locations* were each measured using a single self-created item. Participants were asked to indicate whether their employer allowed them to work from home or other locations outside their employer's premises. If they indicated that they were allowed to do so, they were also asked to indicate for how many hours per week they were allowed to work from home or other locations outside their employer's premises. We divided these numbers by the contractual working hours to get percentage scores that indicated how much of the participants' working time was available to work from home or telework from other locations.

### Results

#### Selection of Items Based on Exploratory Factor Analyses

We conducted exploratory factor analyses in Mplus 8.3 (Muthén and Muthén, 1998–2017) on the initial set of items to shorten the subscales and select items for the final version. Given that the changes in chi-square values between models with different numbers of non-orthogonal factors supported our assumption of four correlated subscales, we relied on the four-factor solution for selecting the final items. Overall, we selected 12 items (i.e., 3 items for each of the 4 subscales) for the final version of the scale that adequately loaded on their respective factor (i.e., factor loading > 0.60), did not show substantial cross-loading (i.e., cross-loadings on other factors <0.10), and logically fitted the relevant construct best (cf. Supplementary Table 1).

#### Reliability and Factor Structure

To check for the internal consistency of the final scale, we calculated Cronbach's alphas for all four subscales (shown in the diagonal of Table 1). Cronbach's alphas ranged between 0.87 and 0.92 and thus were all satisfactory (Nunnally and Bernstein, 1994).

**Table 1:** Descriptive statistics, Cronbach's alphas, and correlations for study 1.

	**1**	**2**	**3**	**4**	**5**	**6**	**7**	**8**	**9**	**10**	**11**	**12**	**13**	**14**
1. Structuring of work tasks^a^	(0.90)													
2. Planning of working times^a^	0.38^*^	(0.87)												
3. Planning of working places^a^	0.20^*^	0.34^*^	(0.92)											
4. Coordinating with others^a^	0.14^*^	0.11	0.33^*^	(0.90)										
5. Information processing^b^	0.39^*^	0.16^*^	0.09	0.41^*^	(0.91)									
6. Problem solving^b^	0.34^*^	0.24^*^	0.32^*^	0.38^*^	0.59^*^	(0.85)								
7. Decision-making autonomy^b^	0.61^*^	0.43^*^	0.22^*^	0.19^*^	0.36^*^	0.44^*^	(0.92)							
8. Work methods autonomy^b^	0.50^*^	0.34^*^	0.26^*^	0.23^*^	0.37^*^	0.51^*^	0.78^*^	(0.91)						
9. Work scheduling autonomy^b^	0.48^*^	0.54^*^	0.11	0.00	0.11^*^	0.21^*^	0.69^*^	0.62^*^	(0.92)					
10. Availability of flextime^c^	0.30^*^	0.77^*^	0.11^*^	0.00	0.12^*^	0.18^*^	0.40^*^	0.33^*^	0.54^*^	(n/a)				
11 Availability of working from home^c^	0.17^*^	0.36^*^	0.13^*^	0.09	0.21^*^	0.20^*^	0.23^*^	0.26^*^	0.27^*^	0.33^*^	(n/a)			
12. Availability of telework from other locations^c^	0.13^*^	0.35^*^	0.30^*^	0.17^*^	0.13^*^	0.21^*^	0.23^*^	0.23^*^	0.25^*^	0.27^*^	0.56^*^	(n/a)		
13. Initiated interdependence^b^	0.11	0.11^*^	0.21^*^	0.26^*^	0.28^*^	0.27^*^	0.14^*^	0.08	0.07	0.00	0.15^*^	0.05	(0.89)	
14. Received interdependence^b^	−0.03	0.12^*^	0.22^*^	0.36^*^	0.22^*^	0.20^*^	0.00	−0.04	0.00	0.05	0.14^*^	0.11	0.59^*^	(0.89)
*n*	303	303	303	303	303	303	303	303	303	303	292	290	303	303
*M*	3.98	2.91	2.32	3.44	4.13	3.45	3.62	3.52	3.41	3.53	10.70	16.63	3.12	2.99
*SD*	0.88	1.34	1.28	1.08	0.82	0.95	0.97	0.95	1.05	1.99	24.45	32.57	1.08	0.99

*Cronbach's alphas are in brackets in the diagonal*;

* *p < 0.05 (two-tailed)*.

a *Subscales from the newly developed cognitive demands of flexible work (CODE) scale*.

b *Subscales from the German translation of the work design questionnaire (Stegmann et al., 2010)*.

c *Measured with self-created items*.

Next, we analyzed the factorial structure of the CODE scale by using confirmatory factor analysis (CFA) techniques based on maximum likelihood estimation in Mplus 8.3 (Muthén and Muthén, 1998–2017). Table 2 shows the factor loadings of the items, as well as the means, standard deviations, and corrected item-total correlations. The four-factor model with the items of each subscale loading on separate factors showed an excellent fit (cf. Hu and Bentler, 1999), as the comparative fit index (CFI) and Tucker-Lewis index (TLI) were above 0.95, the root mean square error of approximation (RMSEA) was below 0.06, and the standardized root mean squared residual (SRMR) was below 0.08 (compare Table 3).

**Table 2:** Item wording, means and standard deviations, corrected item-total correlations, and standardized factor loadings from confirmatory factor analysis.

				**Latent factor**			
**Item wording (translated)**	***M***	***SD***	**CITC**	**1**	**2**	**3**	**4**
**Structuring of work tasks**							
My job requires me to define the individual work steps myself.	3.88	1.02	0.83	0.91			
My job requires me to determine the sequence of my work steps on my own.	3.96	0.99	0.83	0.91			
My job requires me to monitor the progress of my work on my own.	4.10	0.89	0.73	0.77			
**Planning of working times**							
Due to my flexible schedule, I have to decide on my own when to start, pause, and end my workday.	2.74	1.48	0.80		0.89		
Due to my flexible schedule, I have to decide how long I work on which weekdays.	2.75	1.52	0.82		0.93		
Due to my flexible schedule, I have to make sure to plan time for breaks.	3.24	1.50	0.66		0.70		
**Planning of working places**							
At work, I have to plan where to work on certain tasks, because I do not have the same work materials available everywhere.	2.28	1.40	0.83			0.88	
At work, I have to plan where to work on certain tasks, because concentrated work is not possible at every location.	2.29	1.34	0.84			0.90	
At work, I have to plan where to work on certain tasks, because I can execute some tasks better in certain places.	2.40	1.40	0.83			0.88	
**Coordinating with others**							
My job often requires that I coordinate with other people regarding the content of our work.	3.55	1.14	0.79				0.84
My job often requires that I coordinate with other people regarding our schedules.	3.34	2.10	0.82				0.88
My job often requires me to come to an agreement with other people regarding a common approach.	3.44	1.19	0.82				0.89

*CITC, corrected item-total correlation; Analyses based on study 1 (N = 303)*.

**Table 3:** Fit indices for measurement models in and measurement invariance tests across the samples of the three studies.

**Model**	**χ^2^**	***df***	**CFI**	**TLI**	**RMSEA [90% CI]**	**SRMR**	**AIC**	**Δχ^2^**	**Δ*df***	***p***	**ΔCFI**
**Measurement model for study 1^a^**											
Four-factor model	70.84	48	0.991	0.987	0.040 [0.017, 0.058]	0.037	9,478.26				
Best-fitting three-factor model^b^	527.30	51	0.806	0.749	0.176 [0.162, 0.189]	0.117	9,928.73	456.46	3	<0.001	0.185
**Measurement model for study 2**											
Four-factor model	86.52	48	0.983	0.976	0.054 [0.035, 0.072]	0.040	8,850.76				
**Measurement invariance tests across German (study 1) and English (study 2) versions^c^**											
Configural invariance	157.36	96	0.987	0.982	0.047 [0.033, 0.060]	0.038	18,329.02				
Metric invariance	164.91	104	0.987	0.974	0.045 [0.032, 0.058]	0.040	18,320.57	7.55	8	0.479	0.000
Scalar invariance	215.46	112	0.978	0.974	0.057 [0.045, 0.068]	0.047	18,355.12	50.55	8	<0.001	0.009
**Measurement model for study 3**											
Four-factor model	95.85	48	0.985	0.980	0.042 [0.030, 0.055]	0.037	16,245.61				

*CFI, comparative fit index; TLI, Tucker-Lewis index; RMSEA, root mean square error of approximation; CI, confidence interval; SRMR, standardized root mean squared residual; AIC, Akaike information criterion; Study 1 (n = 303), study 2 (n = 274), study 3 (n = 522)*.

a *The best-fitting three factor model is compared to the four-factor model*.

b *Model with the items of structuring of work tasks and planning of working times loading on the same factor*.

c *The metric model is compared to the configural model and the scalar model is compared to the metric model*.

We also compared the four-factor measurement model against different three-factor models, in which we set the items of two subscales to load on the same factor. We calculated six different three-factor models to test all possible combinations of any two subscales loading on the same factor. The best-fitting three-factor model is also shown in Table 3. To compare this model to the four-factor model, we calculated chi-square differences, and because chi-square-difference tests are known to be biased in larger sample sizes, we also calculated CFI differences. A CFI difference between two models of 0.01 or smaller is considered to indicate that both models fit equally well (Cheung and Rensvold, 2002). The best-fitting three-factor model (i.e., the model with the items of structuring of work tasks and planning of working times loading on the same factor) fitted worse than the four-factor model based on a significant chi-square-difference test and showed a CFI difference considerably larger than 0.01 (compare Table 3). Thus, the four-factor structure of the CODE scale was initially supported.

#### Convergent and Divergent Validity

To test for the convergent validity of the CODE scale, we investigated the correlations of the four subscales with other working conditions that were measured with established scales. To make sure that we did not capitalize on chance when testing our hypotheses, we divided the threshold for significance by the number of correlations being tested for the respective hypothesis. When testing Hypothesis 1, the Bonferroni-corrected α was 0.05/8 = 0.00625 because Hypothesis 1 included 8 correlations. When testing Hypotheses 2 through 5, the Bonferroni-corrected α was 0.05/2 = 0.025 because Hypotheses 2 through 5 each included 2 correlations.

All subscales, except planning of working places, showed significant positive associations with information processing (compare Table 1) that remained significant after Bonferroni correction. Moreover, in line with our assumption, all four subscales showed significant positive associations with problem solving (compare Table 1) that again remained significant after Bonferroni correction. Thus, Hypothesis 1 was largely supported.

Further, in line with Hypotheses 2 through 5, structuring of work tasks showed significant positive associations with decision-making autonomy and work methods autonomy; planning of working times showed significant positive associations with work scheduling autonomy and the availability of flextime; planning of working places showed significant positive associations with the availability of working from home and the availability of telework from other locations; and coordinating with others showed significant positive associations with initiated interdependence and received interdependence (compare Table 1). All of these correlations remained significant after Bonferroni correction, with the exception of the correlation between planning of working places and the availability of working from home. Thus, Hypotheses 2, 3, and 5 were fully supported, and Hypothesis 4 was partly supported.

To show that the four subscales were not only significantly related to similar constructs but also measured constructs that were still distinct from these similar constructs (i.e., to test for divergent validity), we conducted a series of confirmatory factor analyses (compare Table 4). For each of the four subscales, we calculated a model that included the focal subscale, the scales for information processing and problem solving, and the other two respective measures that had been used to show convergent validity above. For each subscale, we further estimated four four-factor models that each combined the focal subscale with one of the four other measures in the analysis. The results from the chi-square-difference tests and CFI differences indicated that for all four subscales the five-factor models fitted the data best. This shows that all subscales measured constructs that were empirically separable from similar constructs (compare Table 4).

**Table 4:** Fit indices of models to test whether each subscale measures a construct that is different from other similar constructs.

**Model**	**χ^2^**	***df***	**CFI**	**TLI**	**RMSEA [90% CI]**	**SRMR**	**AIC**	**Δχ^2^**	**Δ*df***	***p***	**ΔCFI**
**Structuring of work tasks vs. information processing, problem solving, decision-making autonomy, and work methods autonomy**											
Five-factor model	244.84	109	0.966	0.958	0.064 [0.053, 0.075]	0.045	10,996.35				
Best-fitting four-factor model^a^	590.99	113	0.882	0.858	0.118 [0.109, 0.128]	0.068	11,334.50	346.15	4	<0.001	0.084
**Planning of working times vs. information processing, problem solving, work scheduling autonomy, and availability of flextime**											
Five-factor model	197.26	94	0.971	0.964	0.060 [0.048, 0.072]	0.047	12,285.70				
Best-fitting four-factor model^b^	300.17	98	0.944	0.932	0.083 [0.072, 0.093]	0.050	12,380.61	102.91	4	<0.001	0.027
**Planning of working places vs. information processing, problem solving, availability of working from home, and availability of telework from other** **locations**											
Five-factor model	102.36	57	0.981	0.974	0.051 [0.035, 0.067]	0.034	13,410.17				
Best-fitting four-factor model^c^	205.58	60	0.939	0.921	0.089 [0.076, 0.103]	0.065	13,507.39	103.22	3	<0.001	0.042
**Coordinating with others vs. information processing, problem solving, initiated interdependence, and received interdependence**											
Five-factor model	216.74	109	0.969	0.962	0.057 [0.046, 0.068]	0.044	12,194.50				
Best-fitting four-factor model^d^	696.99	113	0.834	0.800	0.131 [0.121, 0.140]	0.090	12,666.75	480.25	4	<0.001	0.135

*CFI, comparative fit index; TLI, Tucker-Lewis index; RMSEA, root mean square error of approximation; CI, confidence interval; SRMR, standardized root mean squared residual; AIC, Akaike information criterion; Analyses based on study 1 (N = 303)*.

a *Model with the items of structuring of work tasks and decision-making autonomy loading on the same factor*.

b *Model with the items of planning of working times and availability of flextime loading on the same factor*.

c *Model with the items of planning of working places and availability of telework from other locations loading on the same factor*.

d *Model with the items of coordinating with others and problem solving loading on the same factor*.

### Study 2: English Translation and Criterion Validity

The goal of the second study was to translate the newly created scale for measuring cognitive demands of flexible work from German to English and check for invariance between the two versions. Further, this study aimed to gather information on the criterion validity of the newly developed scale.

Drawing on challenge—hindrance stressor literature (e.g., LePine et al., 2005; Crawford et al., 2010), we assumed that the cognitive demands of flexible work are to be considered challenge demands. In the challenge—hindrance stressor literature, it is asserted that job demands can be differentiated into challenge demands and hindrance demands (e.g., Cavanaugh et al., 2000). Although both types of job demands require effort and are, therefore, associated with strain, their associations with motivational outcomes differ (e.g., Crawford et al., 2010). Challenge demands show positive associations with motivational outcomes, whereas hindrance demands share negative associations with motivational outcomes (e.g., Crawford et al., 2010). These differential relationships with motivation are usually explained by the assertion that individuals are likely to believe that the effort expended in coping with challenge demands will help them to attain work goals and other valued outcomes (e.g., LePine et al., 2005).

We expected the cognitive demands of flexible work to be associated with strain-related and motivational work outcomes. As structuring, planning, and coordinating work will, in most cases, require deliberate cognitive operations, such as information processing, decision-making, or problem-solving, based on action-regulation theory (cf. Zacher and Frese, 2018), we assumed that dealing with them will require cognitive effort. Because the conditions of work vary in flexible work environments, it is difficult for employees to use predefined schemas, learned procedures, or routines for dealing with the cognitive demands of flexible work. Rather, the cognitive demands of flexible work require conscious attention and analysis of the respective situation. Therefore, when dealing with cognitive demands of flexible work, employees expend cognitive effort and experience psychological costs (Hockey, 1997; Zacher and Frese, 2018). In the research on job demands, such psychological costs are usually operationalized with indicators of burnout, such as emotional exhaustion (e.g., feeling burned out or used up from one's work). With regard to motivational work outcomes, we focused on work engagement (i.e., feeling vigorous, dedicated, and absorbed during work). We assumed that the cognitive demands of flexible work would be positively related to work engagement, as the effort invested in these demands would likely be helpful in attaining work goals and provide opportunities to experience competence, autonomy, and connectedness (cf. Gagné and Deci, 2005). We expected that all four subscales of the CODE scale would share positive relationships with emotional exhaustion and engagement (Hypothesis 6).

Further, as previous literature has shown that flexibly organized work tends to blur the boundaries between work and non-work domains (e.g., Spieler et al., 2017), we expected that structuring, planning, and coordinating would also be associated with employees' cognitions during off-job times. Therefore, cognitive demands of flexible work should be associated not only with traditional indicators of strain and motivation but also with indicators of rumination during leisure time. Having to make decisions about how to structure, plan, and coordinate one's work should provide employees with both positive and negative experiences that cognitively occupy them during off-job time. For example, if a person manages to successfully structure a complex work task and create a plan for how to tackle it, they might reflect on this result, even when they are away from their work. Similarly, if they fail to come to an agreement with other people regarding a common approach, employees might be more likely to ruminate on their work experiences. Additionally, it seems rather likely that employees will ponder on unsolved problems that arose from structuring, planning, and coordinating their work and try to solve them during their leisure time. Consequently, we expected that all four subscales of the CODE would share positive relationships with positive work rumination, negative work rumination, and problem-solving pondering (Hypothesis 7).

#### Method

##### Procedure

Data on the English CODE scale were collected using an online questionnaire. The procedure and materials of this study did not undergo examination by an ethics committee, as the measures and procedures of our study followed standard procedures in applied psychological research, and we did not touch on sensitive topics (e.g., sexual orientation). Our protocol fully complied with the ethical principles of psychologists and the code of conduct of the American Psychological Association (2017). Participation was voluntary, and participants were free to withdraw whenever they wanted.

##### Sample

The sample consisted of employees from the United Kingdom working at least 30 contractual hours per week and was recruited via the same certified online panel that had been used in the first study.

In total, 274 employees participated in study 2. About half (49.1%) of the sample was female. The average age of the participants was 41.22 years (*SD* = 10.64); they worked on average 37.53 h per week (*SD* = 3.46), and they had worked on average 7.41 years (*SD* = 6.86) for their current company. About half (47.4%) of the participants had a leadership position, while slightly more than half (58.4%) of the participants held an academic degree.

##### Measures

The items were first translated from German to English by native speakers in both languages using a back-translation method. Participants responded to the translated version of the CODE scale in English. They were asked to indicate on a 5-point scale how the statements of the generated items applied to their work in general (1 = *not at all* to 5 = *fully*).

Emotional exhaustion was measured with 3 items that we selected from the Maslach Burnout Inventory (Schaufeli et al., 1996). Participants were asked to answer the 3 items on a 5-point rating scale (1 = *do not agree at all* to 5 = *fully agree*). A sample item is, “I feel emotionally drained from my work.”

*Work engagement* was measured using the ultra-short version of the Utrecht Work Engagement Scale (Schaufeli et al., 2019). Participants were asked to answer 3 items on a 5-point rating scale (1 = *do not agree at all* to 5 = *fully agree*). A sample item is, “At my work, I feel bursting with energy.”

*Positive work rumination* and *negative work rumination* were assessed by adapting a measure that Frone (2015) initially developed. Participants were asked to answer 3 items for each aspect of work rumination on a 5-point rating scale (1 = *does not apply* to 5 = *fully applies*). We adapted the measure by Frone (2015) for this study by dropping one negative and one positive rumination item and adding the introduction “During my leisure time” to the six retained items. A sample item for positive work rumination is, “During my leisure time, I replay positive work events in my mind.” A sample item for negative work rumination is, “During my leisure time, I think back to the bad things that happened at work.”

*Problem-solving pondering* was measured by adapting a scale that Cropley et al. (2012) initially developed. Participants were asked to answer 4 items on a 5-point rating scale (1 = *does not apply* to 5 = *fully applies*). We adapted the measure by Cropley et al. (2012) for this study by dropping one item and adding the introduction “During my leisure time” to the four retained items to keep their wording similar to the positive and negative work rumination items. A sample item is, “During my leisure time, I find solutions to work-related problems.”

### Results

#### Reliabilities and Factor Structure

To check for internal consistency in the English version, we calculated Cronbach's alphas (shown in the diagonal of Table 5). Cronbach's alphas ranged between 0.84 and 0.91 and thus were all satisfactory (Nunnally and Bernstein, 1994). We also checked the factorial structure of the English version to cross-validate the results from study 1. Again, confirmatory factor analyses showed a good to excellent model fit (compare Table 3; cf. Hu and Bentler, 1999).

**Table 5:** Descriptive statistics, Cronbach's alphas, and correlations for study 2.

	**1**	**2**	**3**	**4**	**5**	**6**	**7**	**8**	**9**
1. Structuring of work tasks^a^	(0.84)								
2. Planning of working times^a^	0.44^*^	(0.87)							
3. Planning of working places^a^	0.43^*^	0.67^*^	(0.91)						
4. Coordinating with others^a^	0.51^*^	0.31^*^	0.42^*^	(0.91)					
5. Emotional exhaustion^b^	0.01	0.01	0.07	0.15^*^	(0.93)				
6. Work engagement^c^	0.35^*^	0.24^*^	0.31^*^	0.34^*^	−0.22^*^	(0.87)			
7. Positive work rumination^d^	0.23^*^	0.28^*^	0.36^*^	0.26^*^	−0.01	0.53^*^	(0.87)		
8. Negative work rumination^d^	0.18^*^	0.15^*^	0.21^*^	0.24^*^	0.48^*^	−0.03	0.33^*^	(0.91)	
9. Problem-solving pondering^e^	0.37^*^	0.30^*^	0.42^*^	0.35^*^	0.19^*^	0.41^*^	0.66^*^	0.58^*^	(0.86)
*n*	269	273	271	272	274	274	274	274	274
*M*	3.47	2.65	2.69	3.53	2.96	3.24	2.69	2.78	2.92
*SD*	1.05	1.28	1.28	1.07	1.16	1.03	0.99	1.10	0.98

*Cronbach's alphas are in brackets in the diagonal*;

* *p < 0.05 (two-tailed)*.

a *Subscales from the newly developed cognitive demands of flexible work (CODE) scale*.

b *Measure adapted from Maslach Burnout Inventory (Schaufeli et al., 1996)*.

c *Measure based on ultra-short version of the Utrecht Work Engagement Scale (Schaufeli et al., 2019)*.

d *Measures adapted from Frone (2015)*.

e *Measure adapted from Cropley et al. (2012)*.

#### Invariance Tests Across Language Versions

To test whether the CODE scale measured the same underlying constructs across both language versions, we conducted a series of measurement invariance tests (cf. Vandenberg and Lance, 2000) across the samples from study 1 (German version) and study 2 (English version). The configural invariance model fit the data well and showed that the four-factor structure (with freely estimated factor loadings and item intercepts for each sample) fitted the combined data from both samples well (cf. Table 3). To additionally test for metric invariance, we set the factor loadings to be equal across the two samples. Both a non-significant chi-square difference and a CFI difference below 0.01 indicated that metric invariance could be assumed. This means that the German and English versions measured the same underlying constructs.

To further test for scalar invariance, we set the factor loadings and item intercepts to be equal across both samples. While the CFI difference was below 0.01, the chi-square-difference test was significant (compare Table 3), which was likely due to chi-square-difference tests often being biased in larger sample sizes (Cheung and Rensvold, 2002). As the model fit indices for the scalar invariant model were still excellent (cf. Hu and Bentler, 1999), the means of the subscales could be compared across the language versions.

#### Criterion Validity

To test the criterion validity of the CODE scale, we interpreted the correlations shown in Table 5. To make sure that we did not capitalize on chance when testing our hypotheses, we divided the threshold for significance by the number of correlations being tested for the respective hypothesis. When testing Hypothesis 6, the Bonferroni-corrected α was 0.05/8 = 0.00625 because Hypothesis 6 included 8 correlations. When testing Hypothesis 7, the Bonferroni-corrected α was 0.05/12 = 0.00416 because Hypothesis 7 included 12 correlations.

The picture regarding the hypothesized association of cognitive demands of flexible work with traditional indicators of strain and motivation was not clear-cut. The correlations of the four subscales with emotional exhaustion were only significant for coordinating with others. However, this correlation was no longer significant after Bonferroni correction. In contrast, the results showed that all four subscales were positively associated with work engagement, even after Bonferroni correction. Thus, Hypothesis 6 was only partly supported.

The predicted associations between the cognitive demands of flexible work and indicators of rumination showed the expected pattern: All four subscales showed significant correlations with positive work rumination, negative work rumination, and problem-solving pondering. All of these correlations remained significant after Bonferroni correction, with the exception of the correlation between planning of working times and negative work rumination. Overall, Hypothesis 7 was largely supported.

## Study 3: Organizational Sample and Differences Between Groups of Employees

The goal of the third study was to test the newly developed scale in an organizational sample with highly flexible work organization. Further, this study aimed to gather information on whether the instrument was capable of reproducing expected differences between relevant groups of employees.

First, we expected employees with leadership positions to show higher cognitive demands of flexible work, as traditionally, structuring, planning, and coordinating work are considered management tasks (e.g., Fayol, 1954). Further, we expected employees with academic degrees to be more exposed to cognitive demands of flexible work than other employees, as—similar to managers—high-skilled professionals usually conduct more cognitively demanding jobs (e.g., Eurofound International Labour Office, 2017). As the increased projectification of organizations also reflects shifts toward more flexible work (e.g., Midler, 1995), we expected employees working mainly on projects to be exposed to more cognitive demands of flexible work than employees mostly working on routine tasks. Finally, we expected that demands for structuring, planning, and coordinating would be higher in employees that frequently used the flextime and flexplace options available to them than in employees that seldomly used flexible work arrangements. In summary, we expected that the cognitive demands of flexible work would be higher in employees that had a leadership position, held an academic degree, worked mainly on projects, used flextime arrangements, and used flexplace arrangements (Hypothesis 8).

### Method

#### Procedure

In study 3, data were again collected using an online questionnaire. The procedure and materials of this study did not undergo examination by an ethics committee, as the measures and procedures of our study followed standard procedures in applied psychological research, and we did not touch on sensitive topics (e.g., sexual orientation). Our protocol fully complied with the ethical principles of psychologists and the code of conduct of the American Psychological Association (2017). Participation was voluntary, and participants were free to withdraw whenever they wanted.

#### Sample

Participants of study 3 were employees working at the headquarters of an Austrian company, where an activity-based flexible office system had been introduced recently. Activity-based flexible office systems are characterized by their offering of different work locations that match the requirements of different tasks and work activities. They are usually open-office environments complemented by open, half-open, and enclosed working locations, without assigned workstations, that provide space for concentrated work, as well as for collaboration and conversation in different areas (Wohlers and Hertel, 2017).

In total, 552 employees participated in study 3. About half (42.3%) of the sample was female. The average age of the participants was 43.63 years (*SD* = 9.88); they worked on average 42.03 h per week (*SD* = 8.27) and had worked 14.09 years (*SD* = 13.44) for their current company. About a quarter (24.7%) of the participants had a leadership position, while about half (49.5%) of the participants held an academic degree.

#### Measures

Participants responded to the CODE scale in German. They were asked to indicate on a 5-point scale how the statements of the generated items applied to their work during the past 3 months (1 = *not at all* to 5 = *fully*).

Information on whether participants had a *leadership position* or an *academic degree* was collected as part of their socio-demographic information. We also asked participants to indicate on a 5-point rating scale to what extent they worked on *project versus routine tasks* (1 = *only project tasks* to 5 = *only routine tasks*), and we measured the *use of flextime* and *use of flexplace* using a self-created item for each (“How often do you organize your working times flexibly?” and “How often do you organize your working places flexibly?,” respectively) on a 5-point rating scale (1 = *never* to 5 = *always*).

## Results

### Reliabilities and Factor Structure

To check for internal consistency in the organizational sample, we calculated Cronbach's alphas (shown in the diagonal of Table 6). Here, Cronbach's alphas ranged between 0.78 and 0.91 and were all satisfactory (Nunnally and Bernstein, 1994). Further, we checked the factorial structure in the organizational sample to cross-validate the results from previous studies. Again, confirmatory factor analyses showed a good to excellent model fit (compare Table 3; cf. Hu and Bentler, 1999).

**Table 6:** Descriptive statistics, Cronbach's alphas, and correlations for study 3.

	**1**	**2**	**3**	**4**	**5**	**6**	**7**	**8**	**9**
1. Structuring of work tasks^a^	(0.88)								
2. Planning of working times^a^	0.27^*^	(0.78)							
3. Planning of working places^a^	0.13^*^	0.14^*^	(0.82)						
4. Coordinating with others^a^	0.22^*^	0.20^*^	0.28^*^	(0.91)					
5. Leadership position^b^	0.28^*^	0.12^*^	0.11^*^	0.16^*^	(n/a)				
6. Academic degree^b^	0.16^*^	0.08	0.12^*^	0.32^*^	0.18^*^	(n/a)			
7. Project (vs. routine) tasks^c^	0.32^*^	0.19^*^	0.25^*^	0.44^*^	0.13^*^	0.31^*^	(n/a)		
8. Use of flextime^b^	0.18^*^	0.45^*^	0.10^*^	0.13^*^	0.10^*^	0.01	0.14^*^	(n/a)	
9. Use of flexplace^b^	0.20^*^	0.26^*^	0.30^*^	0.16^*^	0.14^*^	0.02	0.18^*^	0.45^*^	(n/a)
*n*	552	549	551	551	546	549	550	550	550
*M*	4.11	3.70	2.70	3.68	0.25	0.50	2.83	3.28	2.36
*SD*	0.77	0.98	1.11	0.85	0.43	0.50	0.90	1.02	1.13

*Cronbach's alphas are in brackets in the diagonal*;

* *p < 0.05 (two-tailed)*.

a *Subscales from the newly developed cognitive demands of flexible work (CODE) scale*.

b *Measured with self-created items*.

c *Measured with self-created item and recoded for this table: 1 = only routine tasks to 5 = only project tasks*.

### Differences in Scores Between Groups of Employees

When testing for differences in scores between groups of employees, we used one-tailed *t*-tests, as we expected the differences to be in specific directions (compare Table 7). To make sure that we did not capitalize on chance when testing our hypothesis, we divided the threshold for significance by the number of *t*-tests. That is, when testing Hypothesis 8, the Bonferroni-corrected α was 0.05/20 = 0.0025 because Hypothesis 8 included 20 *t*-tests.

**Table 7:** Comparison of means between different groups in study 3.

	**Leadership position**					**Academic degree**					**Working mainly in projects^a^**				
	**Yes**		**No**			**Yes**		**No**			**Yes**		**No**		
	***M***	***SD***	***M***	***SD***	***t***	***M***	***SD***	***M***	***SD***	***t***	***M***	***SD***	***M***	***SD***	***t***
Structuring of work tasks	4.48	0.62	3.99	0.76	6.77^*^	4.23	0.70	3.99	0.78	3.73^*^	4.41	0.59	3.78	0.86	7.69^*^
Planning of working times	3.92	0.99	3.64	0.96	2.87^*^	3.78	0.99	3.63	0.96	1.82^†^	3.90	0.88	3.44	1.02	4.24^*^
Planning of working places	2.91	1.11	2.63	1.10	2.55^*^	2.84	1.08	2.57	1.12	2.93^*^	3.04	1.03	2.29	1.11	6.15^*^
Coordinating with others	3.92	0.84	3.60	0.84	3.86^*^	3.95	0.79	3.41	0.82	7.83^*^	4.08	0.74	3.11	0.83	10.79^*^
*n*	134–135		408–411			271–272		275–277			194–195		121–123		
	**High flextime use** ^**b**^					**High flexplace use** ^**b**^							
	**Yes**		**No**			**Yes**		**No**					
Structuring of work tasks	4.26	0.74	3.97	0.82	3.51^*^	4.36	0.68	4.00	0.78	3.98^*^					
Planning of working times	4.12	0.83	3.11	1.04	10.32^*^	4.13	0.82	3.54	0.98	5.32^*^					
Planning of working places	2.79	1.14	2.41	1.05	3.26^*^	3.21	1.18	2.47	1.05	5.84^*^					
Coordinating with others	3.78	0.85	3.54	0.86	2.59^*^	3.91	0.81	3.61	0.87	3.02^*^					
*n*	246–247		132–134			92		331–334					

† *p < 0.05 (one-tailed)*,

* *p < 0.05 (two-tailed)*.

a *We recoded the item on project (vs. routine) tasks for these analyses: Yes = only or mainly project tasks; No = only or mainly routine tasks; participants indicating that they worked about the same extent on project tasks as on routine tasks were not categorized into either group*.

b *We recoded the items on use of flextime and use of flexplace for these analyses: Yes = Always or most of the time; No = Never or rarely; participants indicating that they used flextime or flexplace “sometimes” were not categorized into either of the groups*.

As expected, employees with a leadership position scored higher in all four subscales than employees without a leadership position. After Bonferroni correction, the differences between both groups remained, with the exception of the difference in planning of working times no longer being significant. Similarly, employees that held an academic degree scored higher in all four subscales than employees without an academic degree. After Bonferroni correction, the differences between the two groups remained again, with the exception of the difference in the planning of workplaces no longer being significant. Employees working mainly on projects scored higher in all four subscales than employees mainly working on routine tasks, and all group differences remained significant after Bonferroni correction. Employees with high flextime use, as well as employees with high flexplace use, showed higher values on the CODE subscales than employees who did not have high flextime or flexplace use. The differences between these groups remained after Bonferroni correction, with the exception of the difference in coordinating with others no longer being significantly different between employees with high vs. low flextime use. Therefore, overall, Hypothesis 8 was largely supported.

## General Discussion

With workplace flexibility and responsibility being shifted from management to employees, new cognitive demands of flexible work are arising. Yet, despite the increasing prevalence of cognitive demands of flexible work, there was still no instrument available to measure these specific demands. Therefore, the present studies developed and initially tested an instrument to capture the extent of cognitive demands, the CODE scale, that consists of four subscales: (1) structuring of work tasks, (2) planning of working times, (3) planning of working places, and (4) coordinating with others. Based on data from samples of employees in Germany, the United Kingdom, and Austria, the CODE scale proved to be a reliable and valid measure.

Results from confirmatory factor analyses supported the four-factor structure in three separate studies, and measurement invariance tests suggested that even scores can be compared across the English and German versions of the scale. Convergent and divergent validity with information processing and problem solving, as well as with other closely matched constructs, were quite satisfactory overall. Tests for criterion validity indicated that the CODE scale was not significantly associated with emotional exhaustion, although analyses regarding associations with work engagement, positive work rumination, negative work rumination, and problem-solving pondering showed satisfactory results overall.

Because the cognitive demands of flexible work did not show a significant positive relationship with emotional exhaustion in our study, the question arises whether the CODE scale can really be considered a measure of job demands or whether—although we made sure that items were worded in terms of the requirements placed on employees—it represents a measure of job resources. As a reviewer noted, it also seems possible that the CODE scale captures job autonomy more neutrally than established scales that tend to have a rather positive (i.e., self-determined) appraisal of job autonomy. Although providing a more neutral measure of job autonomy might in itself be a relevant contribution to the literature, we think that the CODE scale represents a measure of job demands rather than job resources.

The main difference between job demands and job resources lies in their relationships with indicators of work strain (e.g., Crawford et al., 2010). As job demands are defined as “aspects of the job that require sustained physical or mental effort and are therefore associated with certain physiological or psychological costs (e.g., exhaustion)” (Demerouti et al., 2001, p. 501), they should be positively related with indicators of strain. It has been meta-analytically shown that job demands are positively related to indicators of burnout, whereas job resources are negatively related to indicators of burnout (Crawford et al., 2010). Thus, based on the results indicating that the CODE scale shared no significant relationships with emotional exhaustion, it could be argued that it neither resembles typical job demands nor typical job resources.

However, our study also showed that the cognitive demands of flexible work are positively related to indicators of rumination during leisure time. Research on antecedents of problem-solving pondering suggests that reflecting on work during non-work time may be a by-product of cognitively challenging or complex jobs (Bennett et al., 2016; Dettmers and Bredehöft, 2020). The pattern of associations suggests that the cognitive demands of flexible work do not directly initiate emotional arousal but rather trigger cognitive processes of thinking about work (cf. Cropley and Zijlstra, 2011). These may drain employees' resources if they fail to use adequate strategies to recover from work, as can be seen by the positive association of problem-solving pondering and, especially, negative work rumination with emotional exhaustion in our second study (for a similar result, see, e.g., Bennett et al., 2016). Thus, the positive associations with indicators of rumination during leisure time could be interpreted as an indication that cognitive demands of flexible work promote mental strain. Therefore, one could argue that the CODE scale should be conceived as a measure of job demands rather than job resources.

Overall, we think that the cognitive demands of flexible work can be considered challenge demands (e.g., LePine et al., 2005; O'Brian and Beehr, 2019). Although the relationships with emotional exhaustion were not as expected, the positive relationships with indicators of rumination during leisure time suggest that these demands may trigger cognitive processes that can later lead to other strain-related work outcomes. Moreover, all four subscales consistently showed positive associations with work engagement, suggesting that they were also related to motivational work outcomes. We suggest that future research should identify factors that boost the motivating effects and buffer the potentially adverse effects of cognitive demands of flexible work.

Finally, it should be mentioned that we also tested the CODE scale regarding its ability to differentiate between relevant groups of employees. The results showed that overall cognitive demands of flexible work are higher among employees that have a leadership position, an academic degree, work mainly on projects, use flextime arrangements, and use flexplace arrangements. This also provides information on potential risk groups that practitioners should focus on when analyzing and evaluating jobs in highly flexible work settings.

### Limitations of the Current Studies

Although the CODE scale, developed and initially validated to capture cognitive demands of flexible work, has several advantages (e.g., good reliabilities with only three items per subscale and availability in both German and English), there are also some limitations that need to be considered. In our efforts to initially validate the scale, we relied on cross-sectional data that did not allow us to infer causality from the observed relationships between the constructs. For example, it could be that cognitive demands of flexible work do not promote work engagement but rather that organizations tend to grant highly engaged employees more autonomy and flexibility (Lesener et al., 2019) that also comes with additional demands to structure one's work, plan one's working times and working places, and coordinate one's work with others. However, as the goal of our research was to develop and initially validate a scale to measure cognitive demands of flexible work, we leave it to future research to investigate the potential effects of cognitive demands of flexible work on work outcomes using more complex research designs.

Further, it could be argued that our assumption that structuring, planning, and coordinating work will, in most cases, require action regulation at the intellectual level, as employees confronted with these demands need to monitor and process information and actively solve problems in case of inconsistencies, may hold only under specific conditions. We think it is reasonable to assume that it will be difficult for employees to use predefined schemas, learned procedures, or routines to deal with the cognitive demands of flexible work because the conditions of work vary in flexible work environments. However, it is also likely that employees will manage to reduce their cognitive effort, at least to some extent, if they develop routines for structuring, planning, and coordinating. Previous research suggests that routines at work help to conserve employees' energy because they require less cognitive processing from employees (Ohly et al., 2017). Thus, it seems plausible that the more routines employees have already developed, the less cognitive effort will be required to meet the cognitive demands of flexible work. We suggest that researchers planning to use the CODE scale consider including items that focus on the routinization of the respective demands, and/or they can directly measure the level of cognitive effort associated with those demands (e.g., by including items measuring cognitive load due to the respective demands).

It should be noted that we used the sample of study 1 not only during scale development to select items for the final version of the scale but also for initial construct validation. We acknowledge that conducting exploratory factor analyses for item selection, as well as confirmatory factor analyses for construct validation, in the same sample is problematic because one would expect to obtain a well-fitting model if both analyses are conducted in the same dataset. Yet, we also conducted confirmatory factor analyses in studies 2 and 3, and the results of these analyses supported the hypothesized four-factor structure.

Another limitation of our research is that—although we used samples from three different countries and tested the scale across two languages—all samples were collected in Europe and, thus, are somewhat homogeneous with regard to the cultural background of the participants. In studies 1 and 2, participants worked in a wide range of companies and were therefore likely offered different forms of flexible work arrangements by their employers. In study 3, we recruited a sample working at the headquarters of an Austrian company that had recently introduced an activity-based flexible office system (Wohlers and Hertel, 2017). The newly developed scale worked well in all three studies, with good reliabilities and support for the assumed factor structure across all studies. However, we suggest that future research also examine the properties of the CODE scale in other parts of the world to provide information on its validity in other countries.

Finally, the newly developed scale is limited in its scope of flexible work. We focused on aspects of deregulated work and new forms of indirect control that provide employees with flexibility regarding how, when, where, and with whom they work. By focusing on cognitive demands of flexible work among employees, we did not intend to develop a scale that could also be used for self-employed, freelancing, and gig workers. However, we encourage future research to adapt the CODE scale to groups of workers outside of typical work contracts.

### Directions for Future Research

Apart from translations of the scale to additional languages, its validation in countries with potentially different cultural backgrounds, and its extension to self-employed, freelancing, and gig workers, we expect the newly developed CODE scale to encourage research to be conducted on different aspects of flexible work and their effects on work outcomes. Given that our research showed that cognitive demands of flexible work are associated with positive work rumination, negative work rumination, and problem-solving pondering during leisure time, we think that it is important for future research to investigate how structuring, planning, and coordinating continue to have an effect on employees' cognition, affect, and well-being during leisure time and potentially promote both work-home conflict and work-home enrichment. We assume that the cognitively demanding aspects of flexible work may help to explain previously inconsistent findings of negative or neutral effects of flexible work on employee well-being (Ter Hoeven and van Zoonen, 2015). Moreover, it might be interesting to investigate whether cognitive demands of flexible work can help individuals to learn new skills in structuring, planning, and coordinating that they can apply in other domains. Such transfers of acquired skills and competencies are suggested in various theories of work design (Karasek and Theorell, 1990; Frese and Zapf, 1994) and have already been shown empirically (Kohn and Schooler, 1983).

Further, it might be fruitful to consider different ways of coping with cognitive demands of flexible work. Recent literature on job crafting in the context of flexible work arrangements suggests that individuals' reflection on their past choices of working times and working places might help them to select and adapt options that better fit their time and spatial demands (Wessels et al., 2019). Future research could thus empirically test whether previous experience with flexible working or certain competencies in structuring, planning, and coordinating one's work are helpful in coping with the associated cognitive demands. This could help to expand the dearth of knowledge on the specific skills and competencies that employees need to work effectively in flexible work environments (Charalampous et al., 2019) and build a basis for developing workplace training and interventions.

Finally, we want to encourage future research to investigate intraindividual fluctuations in cognitive demands of flexible work, as well as the processes that link cognitive demands of flexible work with work outcomes, in daily working life. A better understanding of how structuring, planning, and coordinating fluctuate from day to day, as well as how cognitive demands of flexible work unfold their potentially strenuous and motivating effects on a daily basis and what role other variables play in these processes, will help in making recommendations for practitioners regarding what to look out for when evaluating psychosocial risks.

In conclusion, we hope that the newly developed and now initially validated scale will aid the accumulation of scholarly knowledge not only on cognitive demands that employees face in today's increasingly deregulated world of work but also on how the adverse and favorable effects of structuring, planning, and coordinating can be mitigated and boosted, respectively.

## Data Availability Statement

The datasets presented in this article are not readily available because they are based on human subject data and the participants have not been asked for consent to share the datasets publicly. Requests to access the datasets should be directed to the corresponding author Roman Prem, roman.prem@uni-graz.at.

## Ethics Statement

Ethical review and approval was not required for the study on human participants in accordance with the local legislation and institutional requirements. Written informed consent for participation was not required for this study in accordance with the national legislation and the institutional requirements.

## Author's Note

Earlier versions of this paper were presented at the 13th conference of the European Academy of Occupational Health Psychology in Lisbon, Portugal, in September 2018, the 11th conference of the Division of Work, Organizational, and Business Psychology of the German Psychological Association in Brunswick, Germany, in September 2019, and at the 80th Annual Meeting of the Academy of Management that was held as a virtual online conference in August 2020. Preprint versions of this paper are available at: https://doi.org/10.31234/osf.io/mxh75.

## Author Contributions

All authors contributed to the development and initial validation of the cognitive demands of flexible work scale. BK initially had the idea to develop the scale. LU and VB conducted focus groups. RP, BK, LU, and VB generated the initial item pool. RP, BK, and CK planned study 1. RP, LU, VB, and CK planned study 2. BK, LU, and CK planned study 3. RP analyzed the data from all three studies. RP and BK wrote the first draft of the manuscript. All authors have read and edited the manuscript and suggested improvements at several stages during the preparation of the manuscript for submission, as well as during the revision of the manuscript.

## Funding

This research was funded in part by the Austrian Science Fund (FWF) P29408-G29.

## Conflict of Interest

The authors declare that the research was conducted in the absence of any commercial or financial relationships that could be construed as a potential conflict of interest.

## Publisher's Note

All claims expressed in this article are solely those of the authors and do not necessarily represent those of their affiliated organizations, or those of the publisher, the editors and the reviewers. Any product that may be evaluated in this article, or claim that may be made by its manufacturer, is not guaranteed or endorsed by the publisher.
