# Supplementary material for: Development and Initial Validation of a Scale to Measure Cognitive Demands of Flexible Work
Source: Front Psychol. 2021 Sep 10;12:679471. doi: 10.3389/fpsyg.2021.679471 (PMC8460915; doi:10.3389/fpsyg.2021.679471)
Supplement: Supplementary file 1 [file Table_1.DOCX]

Supplementary Material

Development and Initial Validation of a Scale to Measure
Cognitive Demands of Flexible Work

Roman Prem^†*^, Bettina Kubicek^†^, Lars Uhlig, Vera Baumgartner, Christian Korunka

^†^These authors have contributed equally to this work and share first authorship

*** Correspondence:** Dr. Roman Prem: roman.prem@uni-graz.at

# Supplementary Table

The table on the following page shows the four-factor solution from the exploratory factor analysis conducted with the initial item pool of 24 items.

**Table S1.** *Item wording in German, means and standard deviations, and standardized factor loadings from exploratory factor analysis with four non-orthogonal factors on the initial item pool.*

|  |  |  | Factor | | | |
| --- | --- | --- | --- | --- | --- | --- |
| Item (in German) | *M* | *SD* | 1 | 2 | 3 | 4 |
| *Strukturieren von Arbeitsaufgaben* |  |  |  |  |  |  |
| Meine Arbeit erfordert, dass ich die einzelnen Arbeitsschritte selbst definiere.* | 3.88 | 1.02 | **.839** | -.006 | .095 | -.055 |
| Meine Arbeit erfordert, dass ich die Reihenfolge meiner Arbeitsschritte selbstständig festlege.* | 3.96 | 0.99 | **.901** | -.040 | .064 | -.068 |
| Meine Arbeit erfordert, dass ich den Fortschritt meiner Arbeit selbstständig überwache.* | 4.10 | 0.88 | **.836** | -.027 | -.069 | .062 |
| Meine Arbeit erfordert, dass ich eigenständig festlege, wann ich welche Aufgaben erledige. | 3.96 | 1.04 | **.876** | .089 | -.074 | -.002 |
| Meine Arbeit erfordert, dass ich selbstständig komplexe Arbeitsaufträge in einzelne Arbeitsschritte unterteile. | 3.90 | 1.04 | **.724** | .096 | .044 | .051 |
| Meine Arbeit erfordert, dass ich eigenständig entscheide, welche Aufgaben ich vorrangig erledigen muss. | 4.12 | 0.99 | **.836** | .007 | -.004 | .050 |
| *Planen der Arbeitszeiten* |  |  |  |  |  |  |
| Aufgrund meiner flexiblen Zeiteinteilung muss ich selbst festlegen, wann ich meinen Arbeitstag anfange, unterbreche und beende.* | 2.74 | 1.47 | .014 | **.905** | -.053 | -.083 |
| Aufgrund meiner flexiblen Zeiteinteilung muss ich meine Arbeitszeiten so planen, dass ich die vorgegebenen Arbeitsstunden nicht übermäßig unter- oder überschreite. | 2.80 | 1.43 | .014 | **.607** | .171 | -.038 |
| Aufgrund meiner flexiblen Zeiteinteilung muss ich die Zusammenarbeit mit meinen Kolleg/-innen planen. | 2.64 | 1.34 | -.091 | .513 | .214 | .299 |
| Aufgrund meiner flexiblen Zeiteinteilung muss ich selbst entscheiden, wie lange ich an welchen Wochentagen arbeite.* | 2.75 | 1.52 | .019 | **.936** | -.091 | -.007 |
| Aufgrund meiner flexiblen Zeiteinteilung muss ich selbst darauf achten, Zeit für Pausen einzuplanen.* | 3.24 | 1.50 | .096 | **.672** | .070 | -.002 |
| Aufgrund meiner flexiblen Zeiteinteilung muss ich meine Arbeitszeiten so planen, dass sie mit meinen beruflichen Terminen abgestimmt sind. | 2.95 | 1.54 | .061 | **.729** | .183 | .023 |
| *Planen der Arbeitsorte* |  |  |  |  |  |  |
| Ich muss bei meiner Arbeit planen, wo ich welche Tätigkeiten ausführe, da ich nicht an jedem Ort dieselben Arbeitsmaterialien zur Verfügung habe.* | 2.28 | 1.40 | .045 | -.060 | **.894** | -.059 |
| Ich muss bei meiner Arbeit planen, wo ich welche Tätigkeiten ausführe, da nicht an jedem Ort konzentriertes Arbeiten möglich ist.* | 2.29 | 1.34 | .016 | .003 | **.866** | .026 |
| Ich muss die Zusammenarbeit mit meinen Kolleg/-innen planen, da ich nicht immer am selben Ort arbeite. | 2.20 | 1.31 | -.011 | .091 | **.695** | .146 |
| Ich muss bei meiner Arbeit planen, wo ich welche Tätigkeiten ausführe, da ich manche Tätigkeiten an bestimmten Orten besser erledigen kann.* | 2.40 | 1.40 | .040 | -.016 | **.911** | -.043 |
| Ich muss bei meiner Arbeit planen, wo ich mich mit anderen Personen treffen und austauschen kann. | 2.56 | 1.34 | -.011 | .210 | **.683** | .026 |
| Ich muss die Zusammenarbeit mit meinen Kolleg/-innen planen, da meine Kolleg/-innen nicht immer im Büro sind. | 2.60 | 1.31 | -.029 | .252 | **.613** | .078 |
| *Koordinieren mit Anderen* |  |  |  |  |  |  |
| Meine Arbeit erfordert, selbst festzulegen, welche Aufgaben ich erledige und welche ich abgebe. | 3.40 | 1.20 | .296 | .175 | -.020 | .222 |
| Meine Arbeit erfordert, dass ich mich häufig mit anderen Personen inhaltlich abstimme.* | 3.55 | 1.14 | .050 | .032 | -.026 | **.819** |
| Meine Arbeit erfordert, dass ich mich häufig mit anderen Personen zeitlich koordiniere.* | 3.34 | 1.19 | -.006 | -.006 | .023 | **.835** |
| Meine Arbeit erfordert, dass ich mich häufig mit anderen Personen auf ein gemeinsames Vorgehen einige.* | 3.44 | 1.18 | .019 | -.027 | -.004 | **.911** |
| Meine Arbeit erfordert, dass ich mich häufig mit anderen Personen darüber austausche, wie wir mit der Arbeit vorankommen. | 3.32 | 1.19 | .028 | -.012 | -.011 | **.858** |
| Meine Arbeit erfordert, dass ich mich häufig mit anderen Personen darüber abspreche, wer welche Aufgaben übernimmt. | 3.21 | 1.28 | -.015 | -.017 | .108 | **.771** |

*Note*. Factor loadings larger than .600 are in bold. Items included in the final version of the scale are marked with an asterisk.
